# Accuracy of Four Tooth Size Prediction Methods on Malay Population

**DOI:** 10.5402/2012/523703

**Published:** 2012-11-14

**Authors:** Belal Khaled Mahmoud, Saifeddin Hamed I. Abu Asab, Haslina Taib

**Affiliations:** School of Dental Sciences, Universiti Sains Malaysia (USM), 16150 Kubang Kerian, Kelantan, Malaysia

## Abstract

*Objective*. To examine the accuracy of Moyers 50%, Tanaka and Johnston, Ling and Wong and Jaroontham and Godfrey methods in predicting the mesio-distal crown width of the permanent canines and premolars (C + P_1_ + P_2_) in Malay population. *Materials and Methods*. The study models of 240 Malay children (120 males and 120 females) aged 14 to 18 years, all free of any signs of dental pathology or anomalies, were measured using a digital caliper accurate to 0.01 mm. The predicted widths (C + P_1_ + P_2_) in both arches derived from the tested prediction equations were compared with the actual measured widths. *Results*. Moyers and Tanaka and Johnston methods showed significant difference between the actual and predicted widths of (C + P_1_ + P_2_) for both sexes. Ling and Wong method also showed statistically significant difference for males, however, there was no significant difference for females. Jaroontham and Godfrey method showed statistical significant difference for females, but the male values did not show any significant difference. *Conclusion*. For male Malay, the method proposed by Jaroontham and Godfrey for male Thai proved to be highly accurate. For female Malay, the method proposed by Ling and Wong for southern Chinese females proved to be highly accurate.

## 1. Introduction

Crowding and spacing in the mixed dentition should be noted as a large number of cases of malocclusion starts during this stage. These cases may be decreased in severity if treated in the right time [1]. Therefore, it is mandatory to estimate size of the unerupted teeth to propose a good orthodontic treatment plan. Mixed dentition space analysis is considered an essential part of the early orthodontic assessment. By the prediction of the size of unerupted teeth in the mandibular or the maxillary arch, it is possible to calculate the amount of space available for their correct alignment. The treatment plan, based on this calculation, may involve serial extraction, space maintenance, or space regaining.

Many methods have been introduced to predict the size of unerupted teeth [2–5]. Moyers prediction tables and Tanaka and Johnston equations have gained wide acceptance [5]. Moyers was the first to predict the widths of the permanent canines, first and second premolars using the sum of the four lower permanent incisors using probability tables. He designed his tables on data derived from a population of Northern European descent. However, the possibility of secular changes within twenty years of producing Moyers tables led Tanaka and Johnston to do their study by repeating Moyers observation but on a new sample of the same ethnicity. They presented prediction equations which had relatively similar values to Moyers tables but were easier to utilize. However, these methods showed less accuracy to predict the size of unerupted teeth when applied on other populations of different ethnic origins [6–8]. This is because there are genetic [9, 10] and racial [11–13] differences with respect to tooth sizes that should be taken into consideration.

The Malay population is an ethnic group of Austronesian people predominantly inhabiting the Malay Peninsula, the east coast of Sumatra, the coast of Borneo, and the smaller islands in between these locations. To our knowledge, no previous studies studied the tooth size prediction in Malay population. Therefore, the purpose of this investigation is to examine the accuracy of the Moyers [3], Tanaka and Johnston [4], Ling and Wong [14], and Jaroontham and Godfrey [15] methods of prediction the size of unerupted teeth in Malay population and to develop a new predicting method for this specific population if the examined methods proved to be of less accuracy.

## 2. Materials and Methods

A random sample of dental study models of 240 Malay patients (120 males and 120 females) who presented complete eruption of all permanent mandibular and maxillary teeth were obtained from the records of the Orthodontic Department in the School of Dental Sciences, USM. The criteria for selection were based on complete fulfillment of the following.

1. All subjects were Malay.
2. Dental casts had to be of high quality and free of distortions. Subjects had to be free of congenitally missing teeth, extracted teeth, malformed teeth, broken, cracked or chipped teeth, or carious lesions.
3. Subjects' ages ranged from 14 to 18 years to forestall any discrepancies based on proximal wear.

A sliding digital caliper (Mitutoyo Manufacturing Co. Ltd., Tokyo, Japan) with an accuracy of 0.01 mm was used to measure the mesiodistal tooth widths of the mandibular central and lateral incisors. Mandibular and maxillary canines (C), first premolars (P_1_), and second premolars (P_2_) of all four quadrants were also measured. The procedure for measuring the mesiodistal tooth width was performed as described by Hunter and Priest [16]. The caliper beaks were inserted labially, and held occlusally parallel to the long axis of the tooth. The beaks were then closed until they gently contacted the contact points of the tooth. The measurements were made as carefully as possible to avoid any damage to the casts, thus driving false readings. All readings were taken by the same investigator.

Values obtained for the right and left posterior segments were averaged so that there would be one value for the maxillary canine and premolars and one value for the mandibular canine and premolars for each value of the mandibular incisors. The mandibular anterior teeth were measured twice in ten percent of the samples (*n* = 24) by the same investigator in order to assess the intraexaminer reliability. In order to avoid bias, the second measurements were taken after the taking of all the first measurements by two weeks. The correlation between the two readings was very high with intraclass correlation (ICC) = 0.98.

From the four methods of prediction which were used in this study, Tanaka and Johnston, Ling and Wong and Jaroontham and Godfrey used linear regression equation (*y* = *a* + *bx*), where *a* and *b* are the equation constants, *x* is the sum of the lower mandibular teeth, and *y* is the sum of the predicted teeth. However, Moyers used a prediction tables instead of equations. His tables do not provide prediction values of unerupted teeth when the sum of anterior teeth is less than 19.5 mm or more than 25.5 mm. Therefore, we converted the Moyers 50% table into a linear regression equation. The equation constants of each method that have been used in this study are summarized in Table 1.

The summations of mesiodistal width of the anterior teeth of all samples were introduced into the tested equations. The predicted width of (C_1_, P_1_, and P_2_) was compared with the actual width measured on the casts by using paired *t*-test. All statistical analyses have been calculated using SPSS 12.0.1 software.

## 3. Results

Descriptive statistics for summation of the mesiodistal widths of upper canine and premolars U(C + P_1_ + P_2_), lower canine and premolars L(C + P_1_ + P_2_), and the lower incisor (LI) were made for males and females and presented in Table 2. The canine-premolar segments in both arches and the lower anterior teeth were significantly larger in males than females with *P* value <0.001 and <0.05, respectively (*t*-test).

The comparisons between the actual and the estimated sum of each canine–premolar segments (C + P_1_ + P_2_) are presented in Tables 3 and 4 for males and females, respectively. 

In male samples, Moyers at the 50 percentile level and Tanaka and Johnston methods showed significant difference with less accuracy in estimating the size of unerupted teeth in the lower arch, while giving relatively accurate estimation in the upper arch. Ling and Wong equations also were inaccurate in predicting the size of unerupted teeth in both arches. On the other hand, Jaroontham and Godfrey's equations predicted the size of unerupted teeth with high accuracy (*P* = 0.40 for maxilla and *P* = 0.47 for mandible).

In the female sample, Moyers, Tanaka and Johnston and Jaroontham and Godfrey methods showed less accuracy in estimating the size of unerupted teeth in both arches. In contrast, the equation designed for Chinese females by Ling and Wong predicted the size of unerupted teeth accurately in both arches (*P* = 0.06 for maxilla and *P* = 0.29 for mandible).

## 4. Discussion

The presence of crowding in the primary dentition stage increases the probability of crowding occurring in the permanent dentition stage. This is because the arch length available anterior to the mandibular second primary molar does not increase after their eruption. Actually the anterior dental arch length decreases with age [17]. Mixed dentition space analysis is considered an essential part of the early orthodontic assessment. By the prediction of the size of unerupted teeth in the mandibular or the maxillary arch, it is possible to calculate the amount of space available for their correct alignment.

Sexual dimorphism was apparent between Malay males and females in all measured teeth. Males' teeth seemed to have bigger mesiodistal dimension than females. Hence, separate prediction equations are recommended for males and females.

Moyers 50% and Tanaka and Johnston methods, which were designed for northern European populations, showed inaccuracy in predicting the size of unerupted teeth. Nevertheless, the methods of prediction designed for Asian populations showed more accuracy on the Malay population. Although Jaroontham and Godfrey regression equations designed for male Thai seem accurate enough to be applied on the male Malay for both arches (mean difference around 0.08 mm), the regression equation designed for female Thai showed less accuracy when applied on female Malay for both arches. In the same way, one of the southern Chinese equations designed by Ling and Wong showed accuracy, on one sex only when applied on the Malay population. The southern Chinese female equation showed reasonable accuracy to be applied on the female Malay (mean difference around 0.15 mm).

The accurate results obtained by applying prediction equations designed for one population on another population do not necessarily mean that these two populations share the same tooth dimensions. However, it may indicate that these populations share the same correlation between anterior and posterior teeth (C + P_1_ + P_2_).

Initially, our intention was to provide regression equations for the Malay population. Nonetheless, the results showed that accurate results can be obtained by applying other prediction equations on the Malay population. This led us to recommend the use of the male Thai equations for the male Malay, and the female southern Chinese equations for the female Malay.

## 5. Conclusion

1. The methods proposed by Moyers 50% and Tanaka and Johnston proved to be inaccurate when applying on Malay population for both sexes.
2. For Malay male, the method proposed by Jaroontham and Godfrey for male Thai proved to be highly accurate.
  - Upper (C + P_1_ + P_2_) = 13.36 + 0.41 × Sum of lower anterior teeth,
  - Lower (C + P_1_ + P_2_) = 11.92 + 0.43 × Sum of lower anterior teeth,
3. For Malay female, the method proposed by Ling and Wong for female southern Chinese proved to be highly accurate.
  - Upper (C + P_1_ + P_2_) = 10.86 + 0.5 × Sum of lower anterior teeth,
  - Lower (C + P_1_ + P_2_) = 9.85 + 0.5 × Sum of lower anterior teeth.

## Figures and Tables

**Table 1:** Equation constants of tested tooth size prediction methods.

Tested methods		Male		Female	
		a	b	a	b
Moyers 50%	Maxilla	9.8	0.55	9.8	0.55
	Mandible	8.6	0.59	8.6	0.59
Tanaka and Johnston	Maxilla	11	0.5	11	0.5
	Mandible	10.5		10.5	
Ling and Wong	Maxilla	11.50	0.5	10.86	0.5
	Mandible	10.61	0.5	9.85	0.5
Jaroontham and Godfrey	Maxilla	13.36	0.41	11.16	0.49
	Mandible	11.92	0.43	9.49	0.53

**Table 2:** Descriptive statistics for LI, U(C + P_1_ + P_2_), and L(C + P_1_ + P_2_) for male and female samples^a^.

Tooth group	Males		Females		
	Mean (mm)	SD (mm)	Mean (mm)	SD (mm)	*P *value*
LI	23.29	1.55	22.68	1.16	<0.05
U(C + P_1_+ P_2_)	21.87	1.03	21.08	1.05	<0.001
L(C + P_1_ + P_2_)	22.82	1.03	21.99	1.03	<0.001

SD: Standard deviation.

^a^Sample size: 120.

*Significant at *P* < 0.05.

**Table 3:** Comparison between actual and predicted values for each tested method. Male samples^a^.

Actual (mm) (C + P_1_ + P_2_) Mean (SD)	Tested methods		Predicted (mm) (C + P_1_ + P_2_) Mean (SD)	Actual-predicted mean difference^b^ (mm) (95% CI)	*P* value*
Maxilla = 22.82 (1.02)	Moyers 50%	Maxilla	22.71 (0.86)	0.11 (−0.09, 0.32)	0.28
		Mandible	22.36 (0.91)	−0.49 (−0.68, −0.31)	<0.001
	Tanaka and Johnston	Maxilla	22.64 (0.77)	0.18 (−0.02, 0.38)	0.08
		Mandible	22.14 (0.77)	−0.28 (−0.45, −0.1)	<0.01
Mandible = 21.87 (1.03)	Ling and Wong	Maxilla	23.14 (0.77)	−0.32 (−0.52, −0.12)	<0.001
		Mandible	22.25 (0.77)	−0.39 (−0.56, −0.21)	<0.01
	Jaroontham and Godfrey	Maxilla	22.90 (0.63)	−0.09 (−0.28, 0.11)	0.47
		Mandible	21.93 (0.67)	−0.07 (−0.25, 0.11)	0.40

^a^ Sample size: 120.

^b^ Independent *t* test.

*Significant at *P* < 0.05.

**Table 4:** Comparison between actual and predicted values for each tested method. Females samples^a^.

Actual (mm) (C + P_1_ + P_2_) Mean (SD)	Tested methods		Predicted (mm) (C + P_1_ + P_2_) Mean (SD)	Actual-predicted mean difference^b^ (mm) (95% CI)	*P* value*
Maxilla = 21.99 (1.03)	Moyers 50%	Maxilla	22.37 (0.64)	−0.38 (−0.59, −0.17)	<0.001
		Mandible	22.00 (0.69)	−0.91 (−1.11, −0.73)	<0.001
	Tanaka and Johnston	Maxilla	22.34 (0.58)	−0.35 (−0.55, −0.14)	<0.001
		Mandible	21.84 (0.58)	−0.75 (−0.95, −0.56)	0.001
Mandible = 21.08 (1.05)	Ling and Wong	Maxilla	22.2 (0.58)	−0.21 (−0.41, 0.00)	0.06
		Mandible	21.18 (0.58)	−0.10 (−0.30, 0.09)	0.29
	Jaroontham and Godfrey	Maxilla	22.27 (0.57)	−0.28 (−0.49, −0.07)	<0.01
		Mandible	22.51 (0.62)	−0.42 (−0.62, −0.23)	<0.001

^a^Sample size: 120.

^b^Independent *t* test.

*Significant at *P* < 0.05.
